# Physical activity modifies the association between Magnesium Depletion Score and chronic pain: A cross-sectional analysis of nationally representative data

**DOI:** 10.1097/MD.0000000000049994

**Published:** 2026-08-07

**Authors:** Jing Yan, Daming Sui, Zuling Zhong, Xuemei Dai, Wei Wu, Gu Gong, Songbai Luo

**Affiliations:** a Department of Anesthesiology, The General Hospital of Western Theater Command, Chengdu, PR China; b Department of Anesthesiology and Pain, The General Hospital of Western Theater Command, Chengdu, PR China.

**Keywords:** chronic pain, Magnesium Depletion Score, NHANES, physical activity, population-based study

## Abstract

Chronic pain is a common public health concern that substantially impairs quality of life. Magnesium is essential for neuromuscular and inflammatory regulation; yet, population-based evidence linking magnesium depletion to chronic pain remains limited. Moreover, whether physical activity modifies this association is unclear. This study examined the association between the Magnesium Depletion Score (MgDS) and chronic pain among US adults and evaluated the modifying role of physical activity. We conducted a cross-sectional analysis of adults aged ≥18 years from the National Health and Nutrition Examination Survey 1999 to 2004. Participants with complete data on MgDS, chronic pain, physical activity, and covariates were included (n = 1908). Chronic pain was defined as pain lasting at least 3 months, with sensitivity analyses using pain lasting more than 1 year. Survey-weighted logistic regression models were used to estimate odds ratios (ORs) and 95% confidence intervals (CIs), adjusting for demographic, socioeconomic, lifestyle, and clinical factors. Physical activity, assessed as total metabolic equivalent task minutes per week, was categorized into tertiles, and interaction analyses were performed. Higher MgDS was significantly associated with increased odds of chronic pain. In fully adjusted models, each 1-unit increase in MgDS was associated with a 15% higher odds of chronic pain (OR: 1.15, 95% CI: 1.00–1.32; *P* = .047). Compared with participants with MgDS = 0, those with MgDS ≥ 2 had a higher likelihood of chronic pain (OR: 1.58, 95% CI: 1.14–2.18; *P* = .008). Results were consistent in sensitivity analyses. Subgroup analyses showed consistent associations across subgroups. Physical activity significantly modified the association between MgDS and chronic pain (*P* for interaction = .009), with stronger associations observed among individuals in the highest physical activity tertile. Magnesium depletion is independently associated with chronic pain among US adults, and this relationship is modified by physical activity. These findings highlight the importance of considering both nutritional status and physical activity in chronic pain management.

## 1. Introduction

Chronic pain, defined as pain persisting or recurring for more than 3 months, represents a major public health challenge worldwide and is a leading cause of disability, reduced quality of life, and increased healthcare utilization.^[1–3]^ In the United States, chronic pain affects a substantial proportion of adults and imposes significant socioeconomic burdens, highlighting the need to identify modifiable risk factors and preventive strategies.^[4,5]^

Magnesium is an essential mineral involved in numerous physiological processes, including neuromuscular transmission, energy metabolism, inflammatory regulation, and neuronal excitability.^[6,7]^ A growing body of evidence suggests that magnesium plays an important role in pain modulation, primarily through its antagonistic effects on N-methyl-D-aspartate (NMDA) receptors, which are central to pain transmission and central sensitization.^[8,9]^ Experimental and clinical studies have demonstrated that magnesium supplementation may reduce pain intensity and improve functional outcomes in patients with chronic pain, particularly those with neuropathic components or low back pain.^[10,11]^

Despite these mechanistic and clinical insights, population-based evidence linking magnesium status to chronic pain remains limited. Prior epidemiological studies have largely focused on dietary magnesium intake or serum magnesium levels, with inconsistent findings across populations.^[12,13]^ Moreover, magnesium depletion is a multifactorial condition influenced by dietary intake, metabolic disorders, medication use, and lifestyle factors, which may not be adequately captured by single biomarkers or intake measures. The Magnesium Depletion Score (MgDS), a composite index integrating multiple risk factors for magnesium deficiency, has been proposed as a more comprehensive indicator of magnesium depletion status in population studies.

Physical activity is another key lifestyle factor closely related to both magnesium homeostasis and chronic pain. Regular physical activity is known to improve musculoskeletal health, reduce inflammation, and alleviate pain symptoms, while also influencing magnesium requirements, distribution, and losses through sweat and metabolic turnover.^[14–16]^ Emerging evidence suggests that physical activity may interact with nutritional status to shape pain-related outcomes.^[17]^ However, whether physical activity modifies the association between magnesium depletion and chronic pain has not been systematically examined in nationally representative populations.

Therefore, using data from the National Health and Nutrition Examination Survey (NHANES) 1999 to 2004, this study aimed to investigate the association between MgDS and chronic pain among US adults and to evaluate the potential modifying role of physical activity. We hypothesized that higher MgDS would be associated with increased odds of chronic pain and that this association would differ across levels of physical activity.

## 2. Methods

### 2.1. Study design and participants

**Figure 1.**
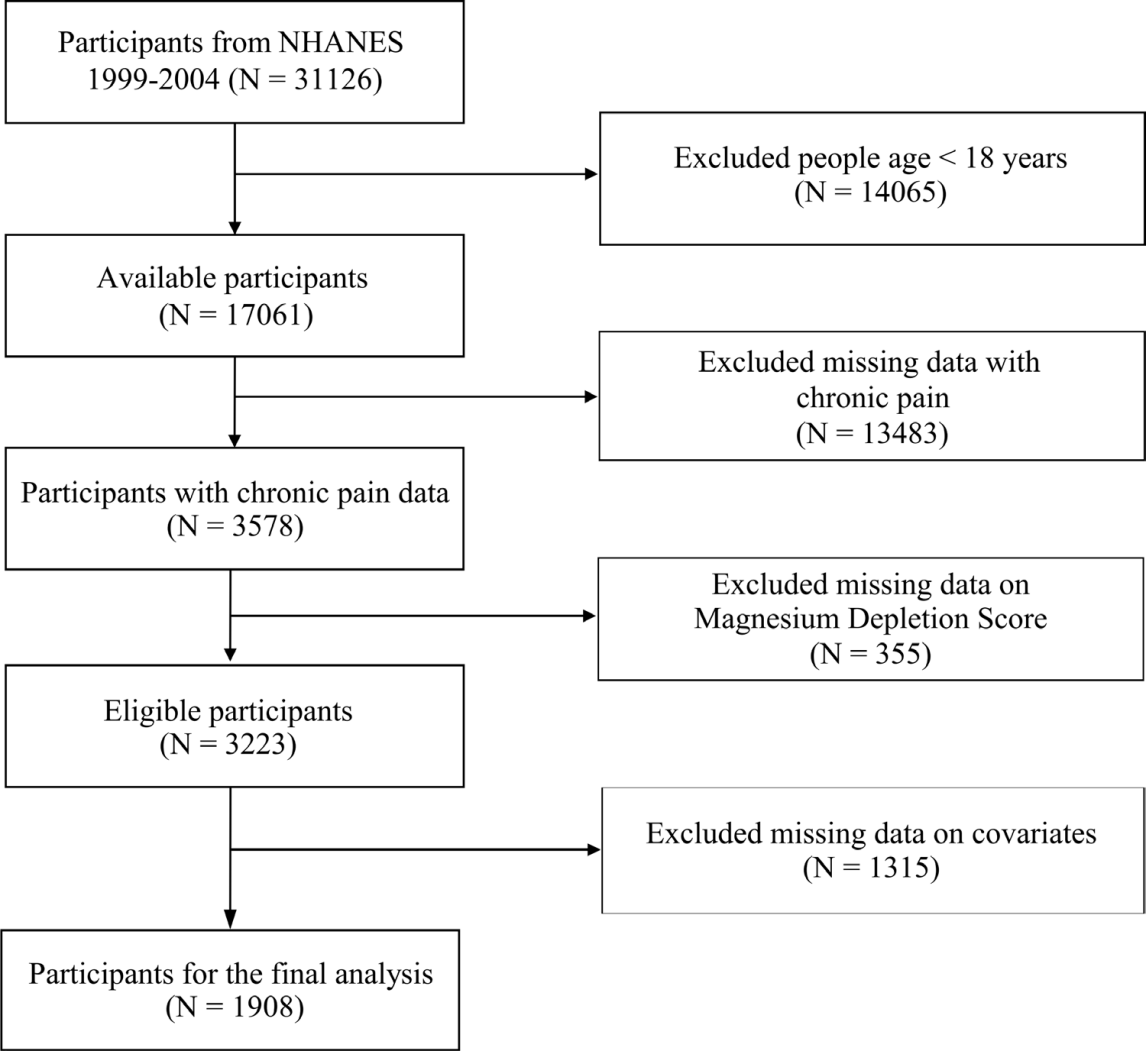
Flowchart of procedures for participant selection and inclusion. NHANES = National Health and Nutrition Examination Survey.

This study was a cross-sectional analysis based on data from the NHANES conducted between 1999 and 2004. These survey cycles were selected because NHANES collected information on pain duration only from 1999 to 2004, making them the only nationally representative data available to define chronic pain based on pain duration. NHANES is a nationally representative survey of the noninstitutionalized civilian US population. Participants aged 18 years or older were considered eligible for the present analysis. The detailed participant selection process is illustrated in Figure 1. After excluding participants younger than 18 years, those with missing chronic pain information, MgDS data, or covariates required for multivariable adjustment, a total of 1908 participants were included in the final analytic sample.

All analyses incorporated NHANES sampling weights to account for the complex survey design. When weighted, the final study population represented approximately 34,624,881 noninstitutionalized US adults, ensuring the national representativeness of the findings.

### 2.2. Ethics statement

The NHANES protocols were approved by the National Center for Health Statistics Ethics Review Board. For the NHANES 1999 to 2004 survey cycles used in the present study, ethical approval was granted under Protocol #98-12. All participants provided written informed consent prior to participation, and all procedures were conducted in accordance with relevant ethical guidelines and regulations. Because the present study was a secondary analysis of publicly available, de-identified data, additional institutional review board approval or informed consent was not required.

### 2.3. Assessment of MgDS

MgDS was used as the primary exposure variable to assess the risk of magnesium depletion. MgDS was calculated by aggregating 4 clinically and biologically relevant factors that have been shown to influence magnesium status, with higher scores indicating a greater risk of magnesium depletion.^[18]^

First, diuretic use was assessed based on self-reported current medication use. Participants who reported current use of diuretics were assigned 1 point. Second, proton pump inhibitor use was determined from prescription medication data, and current proton pump inhibitor users were assigned 1 point. Third, kidney function was evaluated using the estimated glomerular filtration rate (eGFR), which was calculated using the Chronic Kidney Disease Epidemiology Collaboration (CKD-EPI) equation, consistent with the original MgDS development study.^[18]^ Participants with mildly decreased kidney function (60 ≤ eGFR < 90 mL/[minute·1.73 m^2^]) were assigned 1 point, whereas those with moderately to severely decreased kidney function (eGFR < 60 mL/[ minute·1.73 m^2^]) were assigned 2 points. Fourth, alcohol consumption was incorporated into the MgDS, with heavy drinkers assigned 1 point. Heavy drinking was defined as consumption of more than 1 drink per day for women and more than 2 drinks per day for men, in accordance with the US Department of Health and Human Services and the US Department of Agriculture 2015 to 2020 Dietary Guidelines for Americans. The total MgDS was calculated by summing the points from all 4 components, resulting in a higher score reflecting a greater likelihood of magnesium depletion. For statistical analyses, MgDS was examined both as a continuous variable and as a categorical variable. Based on previous applications of this scoring approach, MgDS was categorized as 0, 1, and ≥2 in the primary analysis. Although a higher threshold (MgDS ≥ 3) has also been used in previous studies to indicate greater magnesium depletion risk, the relatively small number of participants with higher MgDS values in our study population could affect the stability of the estimates. Therefore, we additionally performed a sensitivity analysis using an alternative categorization of MgDS (0, 1–2, and ≥3).

### 2.4. Assessment of chronic pain

Chronic pain was assessed using the NHANES Medical Conditions Questionnaire, specifically the variable mpq110, which asked participants: “For how long {have you/has SP} experienced this pain? Would you say….” Response options included less than a month; at least 1 month but <3 months; at least 3 months but <1 year; and >1 year. For the primary analysis, chronic pain was defined as pain lasting at least 3 months, consistent with the definition proposed by the International Association for the Study of Pain. Participants who reported pain lasting at least 3 months but <1 year or >1 year were classified as having chronic pain, whereas those reporting pain lasting <3 months were classified as not having chronic pain. This variable was coded as a binary outcome (yes/no). To assess the robustness of the findings, a sensitivity analysis was conducted using a more stringent definition of chronic pain. In this analysis, chronic pain was defined as pain lasting more than 1 year, and participants reporting shorter pain durations were classified as non-cases. Both outcome definitions were treated as binary variables in subsequent analyses.

### 2.5. Definition of covariates

Covariates were selected a priori based on previous literature and biological plausibility, including demographic characteristics, socioeconomic status, lifestyle factors, and clinical conditions. Demographic variables included age (categorized as 18–29, 30–44, 45–59, and ≥60 years), sex (male or female), and race/ethnicity (non-Hispanic White, non-Hispanic Black, Hispanic, and Other race). Socioeconomic factors included education level (below high school, high school, and above high school), marital status, and poverty income ratio (PIR; categorized as <1.3, 1.3–3.5, and >3.5). Lifestyle-related covariates included smoking status (never, former, or current smoker) and alcohol consumption (never, former, mild, moderate, or heavy drinker). Body mass index (BMI) was calculated as weight in kilograms divided by height in meters squared (kg/m^2^) and categorized as underweight/normal, overweight, or obese. Clinical conditions, including hypertension, hyperlipidemia, and diabetes mellitus, were included as covariates. The detailed definitions and ascertainment of these conditions are provided in Table S1, Supplemental Digital Content 1.

Physical activity was assessed using NHANES physical activity questionnaires and expressed as total metabolic equivalent task minutes per week (PA total MET), calculated based on the frequency, duration, and intensity of reported activities, as described in previous studies.^[19]^ For interaction and stratified analyses, PA total MET was categorized into tertiles, and the cutoff values and distribution details are shown in Table S2, Supplemental Digital Content 2.

### 2.6. Statistical analyses

All statistical analyses were conducted in accordance with the complex sampling design of NHANES. Because data from multiple NHANES cycles (1999–2000, 2001–2002, and 2003–2004) were combined, combined survey weights were constructed following NHANES analytic guidelines. Specifically, a 6-year examination weight was calculated using the following formula: for 1999 to 2002, the weight was calculated as 2/3 × WTMEC4YR, and for 2003 to 2004, it was 1/3 × WTMEC2YR.

Weighted descriptive statistics were used to summarize baseline characteristics of the study population according to chronic pain status. Continuous variables were presented as weighted means with standard errors, and categorical variables were expressed as weighted percentages. Survey-weighted logistic regression models were applied to evaluate the association between MgDS and chronic pain. MgDS was analyzed both as a continuous variable and as a categorical variable (0, 1, and ≥2). Three models were fitted: an unadjusted model; a minimally adjusted model controlling for age, sex, and race/ethnicity; and a fully adjusted model further including BMI, PIR, education level, marital status, smoking status, alcohol consumption, physical activity, hyperlipidemia, hypertension, and diabetes mellitus.

Sensitivity analyses were conducted using an alternative definition of chronic pain (pain lasting more than 1 year) to evaluate the robustness of the primary findings. Subgroup analyses were performed to examine the consistency of associations across predefined strata. To evaluate the potential effect modification by physical activity, interaction analyses between MgDS and physical activity tertiles were conducted. Additionally, the survey-weighted prevalence of chronic pain across categories of MgDS within each physical activity tertile was estimated using survey-weighted means and visualized with 95% confidence intervals (CIs). Multicollinearity among covariates was assessed using variance inflation factors, and all variance inflation factors were <1.2, indicating no evidence of problematic multicollinearity. All analyses were performed using R software (version 4.3.1; R Foundation for Statistical Computing, Vienna, Austria) with appropriate survey and statistical packages.

## 3. Results

### 3.1. Descriptive characteristics of the study population

Weighted descriptive characteristics of the study population stratified by chronic pain status are presented in Table 1. A total of 1908 participants were included in the analysis. Among them, 1076 participants (56.4%) were classified as having chronic pain. Participants with chronic pain had a significantly higher MgDS compared with those without chronic pain (0.90 ± 0.03 vs 0.70 ± 0.03, *P* < .001). The mean level of total physical activity (PA total MET) was higher in the chronic pain group than in the non-chronic pain group, although the difference did not reach statistical significance (1053.3 ± 72.8 vs 904.0 ± 48.9 MET-min/week, *P* = .084).

**Table 1:** Descriptive characteristics of the study population stratified by chronic pain.

Characteristic	Total (N = 1908)	Non-chronic pain group (N = 832)	Chronic pain group (N = 1076)	*P*-values
PA total MET (MET-min/wk)	985.4 ± 47.2	904.0 ± 48.9	1053.3 ± 72.8	.084
Magnesium Depletion Score	0.81 ± 0.02	0.70 ± 0.03	0.90 ± 0.03	**<.001**
Age, n (%)				**<.001**
18–29 yr	280 (15.67)	165 (58.02)	115 (41.98)	
30–44 yr	579 (35.99)	272 (48.21)	307 (51.79)	
45–59 yr	537 (32.62)	234 (43.42)	303 (56.58)	
≥60 yr	512 (15.72)	161 (31.00)	351 (69.00)	
Sex, n (%)				.368
Female	951 (50.36)	398 (43.96)	553 (56.04)	
Male	957 (49.64)	434 (47.03)	523 (52.97)	
Race/ethnicity, n (%)				**.038**
Non-Hispanic White	1224 (81.22)	515 (44.45)	709 (55.55)	
Non-Hispanic Black	282 (6.97)	125 (47.00)	157 (53.00)	
Hispanic	341 (8.27)	171 (56.93)	170 (43.07)	
Other race	61 (3.55)	21 (39.53)	40 (60.47)	
Body mass index, n (%)				.327
Underweight/normal	553 (30.22)	259 (48.66)	294 (51.34)	
Overweight	673 (34.92)	292 (43.74)	381 (56.26)	
Obese	682 (34.87)	281 (44.47)	401 (55.53)	
Marital status, n (%)				**<.001**
Married/living with partner	1249 (68.12)	524 (42.96)	725 (57.04)	
Never married	266 (13.84)	156 (61.25)	110 (38.75)	
Widowed/divorced/separated	393 (18.04)	152 (42.90)	241 (57.10)	
Poverty income ratio, n (%)				**.038**
<1.3	479 (19.85)	185 (40.78)	294 (59.22)	
1.3–3.5	722 (36.09)	302 (42.83)	420 (57.17)	
>3.5	707 (44.06)	345 (49.77)	362 (50.23)	
Education level, n (%)				**.024**
Below high school	151 (3.47)	62 (44.71)	89 (55.29)	
High school	775 (39.86)	311 (41.44)	464 (58.56)	
Above high school	982 (56.67)	459 (48.37)	523 (51.63)	
Alcohol consumption, n (%)				**.029**
Never	202 (9.65)	91 (48.98)	111 (51.02)	
Former	393 (17.29)	136 (36.04)	257 (63.96)	
Mild	627 (33.60)	287 (48.78)	340 (51.22)	
Moderate	301 (17.11)	139 (44.65)	162 (55.35)	
Heavy	385 (22.35)	179 (46.95)	206 (53.05)	
Smoking status, n (%)				**<.001**
Never	843 (44.10)	401 (50.55)	442 (49.45)	
Former	527 (26.40)	224 (45.72)	303 (54.28)	
Current	538 (29.51)	207 (37.69)	331 (62.31)	
Hyperlipidemia, n (%)				.545
No	520 (27.40)	242 (46.76)	278 (53.24)	
Yes	1388 (72.60)	590 (45.00)	798 (55.00)	
Hypertension, n (%)				**.015**
No	1099 (63.14)	517 (48.16)	582 (51.84)	
Yes	809 (36.86)	315 (40.90)	494 (59.10)	
Diabetes mellitus, n (%)				**.004**
No	1591 (87.08)	724 (46.77)	867 (53.23)	
Prediabetes	68 (3.12)	21 (28.16)	47 (71.84)	
DM	249 (9.80)	87 (39.55)	162 (60.45)	

Bold values indicate statistically significant results, defined as a two-sided *P* value <.05.

DM = diabetes mellitus, MET = metabolic equivalent task, PA = physical activity, PA total MET = total physical activity (MET-min/week).

In age-stratified analyses, the distribution of chronic pain status differed significantly across age groups (*P* < .001). A higher proportion of participants aged 45 years and older reported chronic pain compared with younger age groups, whereas a lower proportion of chronic pain was observed among those aged 18 to 29 years (*P* < .001). The distribution of chronic pain status within sex strata did not differ significantly (*P* = .368). In contrast, the proportion of participants reporting chronic pain varied across categories of race/ethnicity (*P* = .038), marital status (*P* < .001), PIR (*P* = .038), education level (*P* = .024), alcohol consumption (*P* = .029), and smoking status (*P* < .001). With respect to clinical characteristics, the proportion of chronic pain differed by hypertension status (*P* = .015) and diabetes mellitus status (*P* = .004). No significant differences in the distribution of chronic pain were observed across BMI categories (*P* = .327) or hyperlipidemia status (*P* = .545).

### 3.2. Association between MgDS and chronic pain

The association between MgDS and chronic pain is shown in Table 2. In survey-weighted logistic regression analyses, higher MgDS was consistently associated with increased odds of chronic pain. When MgDS was analyzed as a continuous variable, each 1-unit increase in MgDS was associated with higher odds of chronic pain in the unadjusted model (odds ratio [OR]: 1.31, 95% CI: 1.17–1.47; *P* < .001). This association remained statistically significant after adjustment for age, sex, and race/ethnicity (OR: 1.15, 95% CI: 1.01–1.32; *P* = .041) and in the fully adjusted model controlling for demographic, socioeconomic, lifestyle, and clinical covariates (OR: 1.15, 95% CI: 1.00–1.32; *P* = .047). When MgDS was categorized, participants with MgDS ≥ 2 had significantly higher odds of chronic pain compared with those with MgDS = 0 in the unadjusted model (OR: 2.11, 95% CI: 1.61–2.76; *P* < .001). This association remained significant after multivariable adjustment (fully adjusted model: OR: 1.58, 95% CI: 1.14–2.18; *P* = .008). In contrast, no significant association was observed for participants with MgDS = 1 compared with those with MgDS = 0 across all models. A significant dose–response relationship between MgDS categories and chronic pain was observed in the unadjusted model (*P* for trend < .001) and persisted after adjustment for potential confounders (*P* for trend = .025 in the fully adjusted model). In the sensitivity analysis using an alternative MgDS categorization (0, 1–2, and ≥3), the association between higher MgDS and chronic pain remained generally consistent with the primary analysis (Table S3, Supplemental Digital Content 3).

**Table 2:** Adjusted association of Magnesium Depletion Score with chronic pain.

Exposure	Unadjusted model	Adjust 1	Adjust 2
	Odds ratio (95% confidence interval) associated with chronic pain; *P*-value		
MgDS (continuous)	1.31 (1.17–1.47); **<.001**	1.15 (1.01–1.32); **.041**	1.15 (1.00–1.32); **.047**
MgDS categories			
Score 0	1 (Ref)	1 (Ref)	1 (Ref)
Score 1	1.03 (0.84–1.25); .772	0.92 (0.75–1.12); .382	0.95 (0.77–1.17); .615
Score ≥ 2	2.11 (1.61–2.76); **<.001**	1.58 (1.15–2.17); **.006**	1.58 (1.14–2.18); **.008**
* P* for trend	**<.001**	**.022**	**.025**

Bold values indicate statistically significant results, defined as a two-sided *P* value <.05.

Unadjusted model: non-adjusted model.

Adjust 1: adjusted for age, sex, and race.

Adjust 2: adjusted for age, sex, race, body mass index, poverty income ratio, education level, marital status, smoking status, alcohol consumption, PA total MET, hyperlipidemia, hypertension, and diabetes mellitus.

MET = metabolic equivalent task, MgDS = Magnesium Depletion Score, PA = physical activity, PA total MET = total physical activity (MET-min/week).

### 3.3. Subgroup and sensitivity analyses

**Figure 2.**
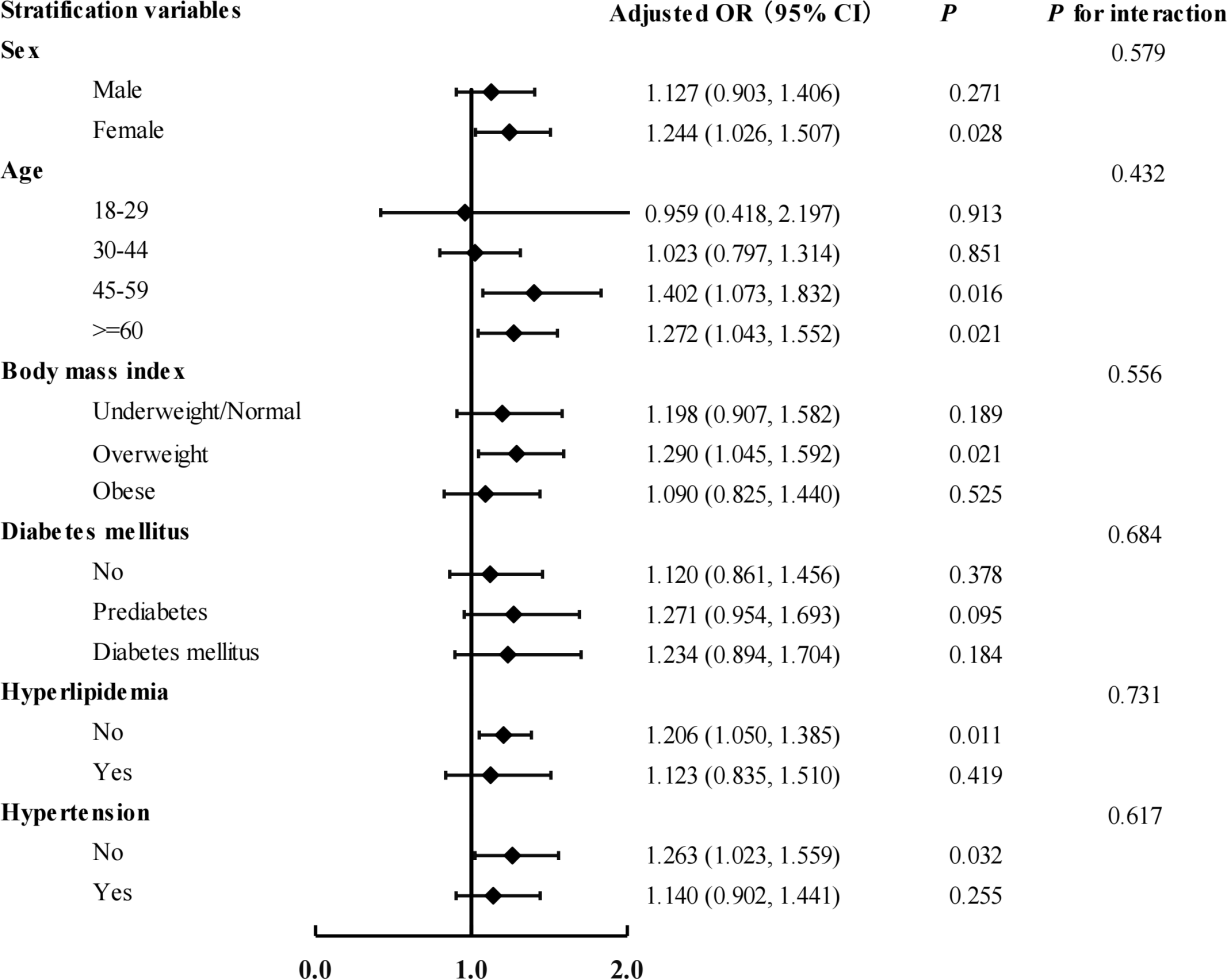
Adjusted association of Magnesium Depletion Score with chronic pain for subgroup analyses. All analyses were adjusted for age, sex, race, body mass index, poverty income ratio, education level, marital status, smoking status, alcohol consumption, hyperlipidemia, hypertension, and diabetes mellitus, but not for the specific stratification variables of interest. CI = confidence interval, OR = odds ratio.

Subgroup analyses were conducted across sex, age, BMI, diabetes mellitus, hyperlipidemia, and hypertension status (Fig. 2). The associations between MgDS and chronic pain were generally consistent across subgroups, with no evidence of substantial heterogeneity.

Sensitivity analyses using an alternative definition of chronic pain (pain lasting more than 1 year) yielded results consistent with the primary analysis (Table 3). Higher MgDS remained associated with increased odds of chronic pain, and a significant trend across MgDS categories persisted after multivariable adjustment.

**Table 3:** Sensitivity analysis was conducted by redefining chronic pain as pain lasting more than 1 year.

Exposure	Unadjusted model	Adjust 1	Adjust 2
	Odds ratio (95% confidence interval) associated with chronic pain; *P*-value		
MgDS (continuous)	1.27 (1.14–1.41); **<.001**	1.17 (1.03–1.31); **.013**	1.19 (1.04–1.36); **.014**
MgDS categories			
Score 0	1 (Ref)	1 (Ref)	1 (Ref)
Score 1	1.02 (0.83–1.25); .834	0.95 (0.76–1.17); .613	0.99 (0.78–1.27); .956
Score ≥ 2	1.88 (1.42–2.49); **<.001**	1.55 (1.13–2.13); **.008**	1.63 (1.14–2.32); **.010**
* P* for trend	**<.001**	**.020**	**.019**

Bold values indicate statistically significant results, defined as a two-sided *P* value <.05.

Unadjusted model: non-adjusted model.

Adjust 1: adjusted for age, sex, and race.

Adjust 2: adjusted for age, sex, race, body mass index, poverty income ratio, education level, marital status, smoking status, alcohol consumption, PA total MET, hyperlipidemia, hypertension, and diabetes mellitus.

MET = metabolic equivalent task, MgDS = Magnesium Depletion Score, PA = physical activity, PA total MET = total physical activity (MET-min/week).

### 3.4. Interaction between physical activity and MgDS

The interaction between physical activity and MgDS in relation to chronic pain is presented in Table 4. A statistically significant interaction between MgDS categories and physical activity tertiles was observed (*P* for interaction = .009). In stratified analyses, higher MgDS was associated with increased odds of chronic pain in the lowest and highest physical activity tertiles, but not in the middle tertile. Specifically, participants with MgDS ≥ 2 had significantly higher odds of chronic pain in the lowest physical activity tertile (OR: 1.87, 95% CI: 1.04–3.37; *P* = .039) and an even stronger association in the highest physical activity tertile (OR: 3.55, 95% CI: 1.81–6.97; *P* = .001).

**Table 4:** Adjusted association of Magnesium Depletion Score with chronic pain in different PA total MET subgroups.

Variables	Odds ratio (95% CI); *P*-value*	*P* for interaction
PA total MET tertile 1 (lowest)		**.009**
MgDS categories		
Score 0	1 (Ref)	
Score 1	0.83 (0.57–1.21); .303	
Score ≥ 2	1.87 (1.04–3.37); **.039**	
PA total MET tertile 2		
MgDS categories		
Score 0	1 (Ref)	
Score 1	0.91 (0.56–1.49); .701	
Score ≥ 2	0.74 (0.37–1.41); .328	
PA total MET tertile 3 (highest)		
MgDS categories		
Score 0	1 (Ref)	
Score 1	1.26 (0.74–2.16); .371	
Score ≥ 2	3.55 (1.81–6.97); **.001**	

Bold values indicate statistically significant results, defined as a two-sided *P* value <.05.

CI = confidence interval, MET = metabolic equivalent task, MgDS = Magnesium Depletion Score, PA = physical activity, PA total MET = total physical activity (MET-min/week).

* The analysis was adjusted for age, sex, race, body mass index, poverty income ratio, education level, marital status, smoking status, alcohol consumption, hyperlipidemia, hypertension, and diabetes mellitus.

**Figure 3.**
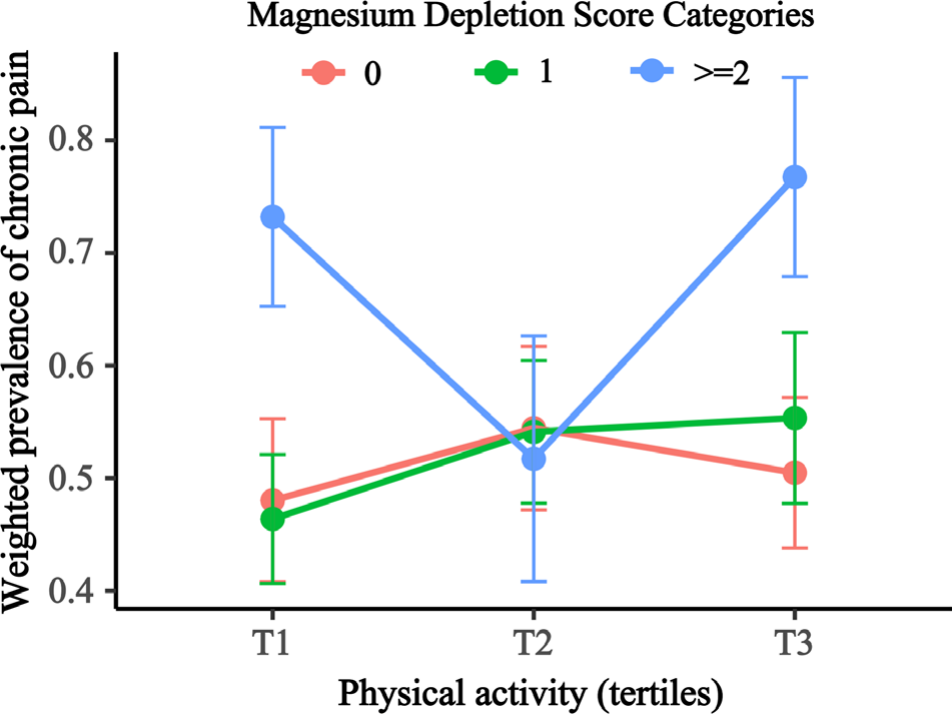
Weighted prevalence of chronic pain across tertiles of total physical activity (MET-min/week) by Magnesium Depletion Score categories among US adults. Points represent weighted prevalence estimates, and error bars indicate 95% confidence intervals. MET = metabolic equivalent task.

Consistent with these results, Figure 3 displays the survey-weighted prevalence of chronic pain across physical activity tertiles stratified by MgDS categories. In the lowest and highest physical activity tertiles, the prevalence of chronic pain increased with higher MgDS categories, whereas no significant differences in chronic pain prevalence were observed across MgDS categories in the middle physical activity tertile.

## 4. Discussion

In this nationally representative study of US adults, a higher MgDS was independently associated with greater odds of chronic pain. This association remained consistent after comprehensive adjustment for demographic, socioeconomic, lifestyle, and clinical factors. Together, these findings indicate that magnesium depletion, as captured by a composite risk score, is a robust correlate of chronic pain in the general population. A key finding of this study is that physical activity significantly modified the association between MgDS and chronic pain. Higher MgDS was associated with increased odds of chronic pain among individuals with low and high levels of physical activity, whereas no clear association was observed among those with moderate levels of physical activity. Notably, the association between MgDS ≥ 2 and chronic pain was strongest in the highest physical activity tertile. These results suggest that the relationship between magnesium depletion and chronic pain may differ across physical activity levels, underscoring the importance of considering interactions between nutritional status and lifestyle factors.

Consistent with prior literature, our findings support an inverse association between magnesium status and chronic pain while extending earlier NHANES-based work that relied mainly on dietary magnesium intake estimates. Higher dietary magnesium intake was associated with lower odds of chronic pain after multivariable adjustment, but intake-based metrics may not fully reflect “true” deficiency risk because they do not capture interindividual differences in absorption, renal handling, or medication-related magnesium loss.^[12]^ One systematic review concluded that magnesium shows analgesic signals in some chronic pain phenotypes (e.g., neuropathic pain, migraine, and complex regional pain syndrome), yet overall efficacy was equivocal due to small samples and heterogeneity.^[20]^ Against this background, using the MgDS, which integrates determinants of magnesium homeostasis, may provide a more clinically relevant characterization than serum magnesium alone; methodological work has shown that MgDS improves the identification of magnesium deficiency and can stratify risk in NHANES, with higher scores associated with systemic inflammation and adverse outcomes.^[18]^

Mechanistically, magnesium is a physiological NMDA-receptor antagonist and may influence central sensitization, an important pathway in chronic pain, while also interacting with calcium channels and related signaling.^[21]^ Magnesium deficiency has additionally been linked to pro-inflammatory activation pathways such as nuclear factor kappa-light-chain-enhancer of activated B cells and NMDA-related processes, and broader reviews describe magnesium as a modulator of inflammatory responses.^[22,23]^ Importantly, our observation that physical activity modifies the MgDS–pain association aligns with exercise physiology evidence that strenuous activity can redistribute magnesium and increase urinary and sweat losses, potentially raising requirements in vulnerable individuals, and with broader reviews linking magnesium status to muscle function and performance as activity demands increase.^[17,24]^ Finally, an updated meta-analysis suggests that magnesium supplementation can reduce C-reactive protein, supporting a plausible inflammation-related pathway connecting magnesium status with pain vulnerability.^[25]^ Together, these data suggest that discrepancies across prior studies may partly reflect how magnesium status is operationalized and whether lifestyle-related effect modification, such as physical activity, is explicitly tested.

This study used nationally representative NHANES data, improving generalizability to US adults. We captured magnesium-related risk using MgDS (a composite indicator of depletion risk) rather than relying only on dietary intake or a single biomarker, and we formally tested effect modification by physical activity to explore heterogeneity in the magnesium–pain association. However, this study also has several limitations that should be noted. The cross-sectional design prevents causal inference and leaves room for reverse causation (pain may reduce activity and influence diet/medication use). Chronic pain and physical activity were self-reported, so misclassification and recall bias are possible. Residual confounding may remain (e.g., pain phenotype/severity, mental health, sleep, medication use, and comorbidities), and single-time measurements may not reflect long-term magnesium status, activity patterns, or pain trajectories. In addition, chronic pain represents a heterogeneous group of conditions with potentially distinct underlying mechanisms. NHANES 1999 to 2004 does not provide detailed information on pain location, severity, or specific pain phenotypes. Therefore, future studies incorporating more comprehensive pain characterization are needed to determine whether the association between magnesium depletion and pain differs across specific pain conditions. Additionally, because MgDS is a composite score, individuals with the same MgDS value may have different combinations of contributing factors and potentially distinct biological profiles. Although subgroup analyses based on specific MgDS component combinations would be informative, such analyses were limited by the small sample size of certain combinations in the present study. Future studies with larger populations are needed to determine whether specific MgDS components or combinations are differentially associated with chronic pain.

These findings may also have practical implications for chronic pain prevention and management in the general population, particularly because both magnesium status and physical activity are modifiable. From a lifestyle perspective, exercise and physical activity programs are widely recommended for chronic pain, and an overview of Cochrane Reviews suggests that although effects on pain severity are not uniformly consistent and the evidence quality is often low, exercise tends to improve physical function and quality of life with few reported harms.^[14]^ In parallel, our results raise the possibility that evaluating magnesium depletion risk – rather than dietary intake alone – could help identify subgroups in whom optimizing magnesium status might be particularly relevant, especially among individuals who are physically active and therefore may face higher physiological demand and losses. Clinically and in public health settings, MgDS could be explored as a pragmatic risk-stratification tool to guide nutritional assessment and counseling, emphasizing magnesium-rich dietary patterns and addressing reversible contributors to depletion, while avoiding overinterpretation of cross-sectional associations as treatment effects.

## 5. Conclusion

In this nationally representative NHANES analysis, a higher magnesium depletion risk (MgDS) was associated with a greater likelihood of chronic pain, and this relationship differed by physical activity level. These findings suggest that magnesium depletion may be a relevant, potentially modifiable factor linked to the burden of chronic pain, and that physical activity may shape the strength of this association. Prospective studies and intervention trials are needed to clarify directionality and to determine whether improving magnesium status, particularly among physically active individuals at higher depletion risk, can help reduce chronic pain risk.

## Acknowledgments

We sincerely appreciate the hard work and dedication of the NHANES staff and the principal investigators. We used GPT (OpenAI) to assist with language polishing of the initial draft. We acknowledge the support of the Western Theater Command General Hospital Management Research Project (Grant No. 2024-YGLC-B18).

## Author contributions

**Conceptualization:** Jing Yan, Daming Sui, Songbai Luo.

**Investigation:** Jing Yan, Daming Sui, Zuling Zhong, Xuemei Dai, Wei Wu, Gu Gong, Songbai Luo.

**Methodology:** Jing Yan, Zuling Zhong, Xuemei Dai, Wei Wu, Gu Gong.

**Writing – original draft:** Jing Yan.

**Writing – review & editing:** Jing Yan, Daming Sui, Zuling Zhong, Xuemei Dai, Wei Wu, Gu Gong, Songbai Luo.






