# Supplementary material for: Physical activity modifies the association between Magnesium Depletion Score and chronic pain: A cross-sectional analysis of nationally representative data
Source: Medicine (Baltimore). 2026 Aug 7;105(32):e49994. doi: 10.1097/MD.0000000000049994 (PMC13456714; doi:10.1097/MD.0000000000049994)
Supplement: Supplementary file 1 [file medi-105-e49994-s001.docx]

**Table S1**. The detailed overview of how we obtained information on hypertension, hyperlipidemia, and diabetes.

| **Hypertension** | The inclusion of hypertension was determined by considering both the results of a questionnaire survey and three separate blood pressure measurements taken from the participants. The participants were asked the following questions: " {Have you/Has SP} ever been told by a doctor or other health professional that {you/s/he} had hypertension, also called high blood pressure? Because of {your/SP's} (high blood pressure/hypertension), {have you/has s/he} ever been told to . . . take prescribed medicine?” And Hypertension was also confirmed if the blood pressure measurements exceeded 140/90 mmHg. |
| --- | --- |
| **Hyperlipidemia** | The information regarding hyperlipidemia is primarily obtained based on the following criteria  1. High Triglycerides (TG): TG levels greater than or equal to 150 mg/dL.  2. High Total Cholesterol (TC): TC levels greater than or equal to 200 mg/dL [5.18 mmol/L].  3. Elevated Low-density lipoprotein (LDL)-Cholesterol: LDL cholesterol levels greater than or equal to 130 mg/dL [3.37 mmol/L].  4. Low high-density lipoprotein (HDL)-Cholesterol: In males, HDL cholesterol levels below 40 mg/dL [1.04 mmol/L]. In females, HDL cholesterol levels below 50 mg/dL [1.30 mmol/L].  5. The use of lipid-lowering medications. |
| **Diabetes mellitus (DM)** | The diagnostic criteria for diabetes are:  1. doctor told you have diabetes ({Other than during pregnancy, {have you/has SP}/ {Have you/Has SP}} ever been told by a doctor or health professional that {you have/{he/she/SP} has} diabetes or sugar diabetes?),  2. glycohemoglobin HbA1c (%) >= 6.5,  3. fasting glucose (mmol/l) >= 7.0,  4. random blood glucose (mmol/l) >= 11.1,  5. two-hour OGTT blood glucose (mmol/l) >= 11.1,  6. Use of diabetes medication or insulin,  Prediabetes: impaired fasting glycaemia and impaired glucose tolerance. |
