# Supplementary material for: Physical activity modifies the association between Magnesium Depletion Score and chronic pain: A cross-sectional analysis of nationally representative data
Source: Medicine (Baltimore). 2026 Aug 7;105(32):e49994. doi: 10.1097/MD.0000000000049994 (PMC13456714; doi:10.1097/MD.0000000000049994)
Supplement: Supplementary file 2 [file medi-105-e49994-s002.docx]

**Table S2**. Details of PA total MET division.

| **Variables** | **Division details** | **Corresponding range** | **Frequency** | **Percentage** |
| --- | --- | --- | --- | --- |
| **PA total MET** | T1 | [0.933, 252] | 696 | 36.48% |
|  | T2 | (252, 817] | 576 | 30.19% |
|  | T3 | (817, 18928] | 636 | 33.33% |

**Abbreviations**: PA total MET, Total physical activity (MET-min/week).
