# Supplementary material for: Physical activity modifies the association between Magnesium Depletion Score and chronic pain: A cross-sectional analysis of nationally representative data
Source: Medicine (Baltimore). 2026 Aug 7;105(32):e49994. doi: 10.1097/MD.0000000000049994 (PMC13456714; doi:10.1097/MD.0000000000049994)
Supplement: Supplementary file 3 [file medi-105-e49994-s003.docx]

**Table S3*.*** Sensitivity analysis of the association between magnesium depletion score and chronic pain using an alternative MgDS categorization (0, 1–2, and ≥3).

| **Exposure** | **Unadjusted model** | **Adjust 1** | **Adjust 2** |
| --- | --- | --- | --- |
|  | Odds ratio (95% confidence interval) associated with chronic pain; *P*-value | | |
| **MgDS Categories** |  |  |  |
| Score 0 | 1 (Ref) | 1 (Ref) | 1 (Ref) |
| Score 1-2 | 1.25 (1.02, 1.51); **0.029** | 1.05 (0.86, 1.28); 0.628 | 1.08 (0.87, 1.33); 0.473 |
| Score >=3 | 1.98 (1.11, 3.52); **0.021** | 1.28 (1.15, 2.29); **0.016** | 1.25 (1.03, 2.12); **0.018** |
| *P* for trend | **0.002** | **0.017** | **0.025** |

Unadjusted model: non-adjusted model.

Adjust 1: Adjust for age, sex, race.

Adjust 2: Adjust for age, sex, race, body mass index, poverty income ratio, education levels, marital status, smoking status, alcohol consumption, PA total MET, hyperlipidemia, hypertension, and diabetes mellitus.

**Abbreviations**: MgDS, Magnesium Depletion Score.
